# A New Chaotic Image Encryption Algorithm Based on Transversals in a Latin Square

**DOI:** 10.3390/e24111574

**Published:** 2022-10-31

**Authors:** Honglian Shen, Xiuling Shan, Ming Xu, Zihong Tian

**Affiliations:** 1 School of Mathematical Sciences, Hebei Normal University, Shijiazhuang 050024, China; 2 Department of Mathematics and Computer Science, Hengshui University, Hengshui 053000, China; 3 Department of Mathematics and Physics, Shijiazhuang Tiedao University, Shijiazhuang 050043, China

**Keywords:** image encryption, chaotic, Latin square, transversals, *n*-transversal

## Abstract

In this paper, a new combinatorial structure is introduced for image encryption, which has an excellent encryption effect on security and efficiency. An *n*-transversal in a Latin square has the function of classifying all the matrix’s positions, and it can provide a pair of orthogonal Latin squares. Employing an *n*-transversal of a Latin square, we can permutate all the pixels of an image group by group for the first time, then use two Latin squares for auxiliary diffusion based on a chaotic sequence, and finally, make use of a pair of orthogonal Latin squares to perform the second scrambling. The whole encryption process is “scrambling–diffusion–scrambling”. The experimental results indicated that this algorithm passed various tests and achieved a secure and fast encryption effect, which outperformed many of the latest papers. The final information entropy was very close to 8, and the correlation coefficient was approximately 0. All these tests verified the robustness and practicability of the proposed algorithm.

## 1. Introduction

In recent years, network communication has developed very rapidly, and a large amount of public or private image information is transferred via the public Internet. How to transmit a great deal of image information safely and efficiently has become an increasingly important issue. Image encryption is the main solution. Digital image encryption is a new and relatively independent branch of computer cryptography and a research hot spot in the field of information security. Unlike ordinary text information, a digital image has a massive amount of data, a strong correlation between pixels, and other particularities, which make the traditional methods DES, IDEA, and RSA inappropriate. Therefore, various image encryption algorithms have been put forward in the last few years.

Chaos-based encryption algorithms play an important role in existing image encryption algorithms [1,2,3]. Some qualities of a chaotic system such as sensitivity to initial values, parameter sensitivity, ergodicity, etc., make it particularly appropriate to perform image encryption. However, there are some disadvantages in chaotic systems, such as being defined on a set of real numbers, accompanied by short-period phenomena, local linearity, and uneven distribution, and requiring discretization when used [4]; therefore, they are vulnerable to chosen plaintext attacks or known plaintext attacks. Accordingly, more and more high-dimensional chaotic systems [5,6,7] have been applied, along with increasing complexity and unpredictability. The higher the dimension of the chaotic system, the more computation is required. Hence, many new different techniques have been used in image encryption algorithms, including one-time keys [8], DNA coding [9,10,11], genetic manipulation [12,13,14], compressive sensing [15,16], semi-tensor product theory [17,18], finite-precision error [19], natural interval extensions [20], fractal sorting matrices [21], and so on.

Recently, many combinatorial design structures have been applied in cryptography, such as Latin squares [5,22,23,24,25], Latin cubes [6,26,27,28], the Hadamard matrix [29], etc. In particular, the Latin square is the most used. A Latin square defined on a finite integer set *S* is a square matrix, having uniformity for the same number of occurrences of each element in *S*, and the total number of Latin squares is also very large. These characteristics of Latin squares are very suitable for image encryption, so some algorithms according to Latin squares have been put forward. As early as 1949, Shannon pointed out that a perfect password can be expressed by a Latin square in his classic paper [30]. Wu et al. proposed an image encryption scheme by using Latin squares [22]. In this paper, a Latin square was used to generate a one-dimensional mapping for the scrambling process. However, the scrambling efficiency of this algorithm is low and it is vulnerable to attacks. Other algorithms that use Latin squares have the same problem [5]. Then, some algorithms using a pair of orthogonal Latin squares appeared [23,24,25], which can directly generate a two-dimensional mapping, instantly increasing the scrambling efficiency. In addition, these Latin squares can provide pseudo-random sequences for the diffusion process. For example, Xu et al. generated a self-orthogonal Latin square (SOLS) and proposed a new algorithm for image encryption [24]. The SOLS and its transpose form a pair of orthogonal Latin squares, and the SOLS can provide a pseudo-random sequence for the diffusion process. The experimental results showed that this algorithm is safe and highly efficient. The entropy value of the encrypted Lena image reached 7.997, and the correlation coefficient was small. The Latin cube is a kind of complex structure in combinatorial design, and the Latin cube contains several Latin squares. It is more widely used in color image encryption algorithms or grayscale images represented by a bit matrix. Xu et al. put forward a new image encryption scheme by using a 3D bit matrix and orthogonal Latin cubes [26]. Each original image was decomposed into a three-dimensional bit matrix, and a pair of orthogonal Latin cubes was used, not only for confusion, but also for diffusion, which proved that the algorithm is highly safe and efficient. The same as the algorithm in [27], the orthogonality of the 3D Latin cube was fully utilized. In 2021, Hua et al. designed a new CIEA using orthogonal Latin squares and 2D-LSM for color image encryption and realized point-to-point permutation and the random distribution of the pixels in a plain image [6]. The algorithm in [28] also makes full use of the orthogonality of a group of Latin cubes, and the images were transformed into one or several cubes.

As can be seen from the above discussion, the Latin cube is suitable for more complex situations. For grayscale images, the orthogonality and uniformity of Latin squares have better performance. Therefore, in this paper, we propose a novel chaos-based image encryption algorithm according to transversals in a Latin square. For a Latin square of order *n*, there exist plenty of *n*-transversals. Employing an *n*-transversal, we can divide all $n^{2}$ positions into *n* mutually disjoint groups, then permutate the pixels of the image group by group in the first round of substitution. We can also define two new Latin squares according to the *n*-transversal, which can be used for auxiliary diffusion on the basis of a chaotic sequence. Finally, a pair of orthogonal Latin squares is reused for the second scrambling. The whole structure is “scrambling–diffusion–scrambling”. The simulation results showed that the proposed method outperformed many of the latest papers in terms of some statistical safety indicators. The main contributions of this article are presented as follows:

- An *n*-transversal in a Latin square is used for image encryption. This combinatorial structure has two functions: classify all the positions of a square and generate two new orthogonal Latin squares.
- We permutated the pixels of the image group by group in the first round of substitution according to an *n*-transversal. Two suitable Latin squares were used for auxiliary diffusion, and another pair of orthogonal Latin squares was also used for the second scrambling.
- The experimental results indicated that this algorithm can make full use of the new combinatorial structure. It passed various tests and had a high security level and a fast speed. The comparison results indicated that it outperformed many of the latest papers.

In the rest of this article, some primary definitions and conclusions are introduced in Section 2. Section 3 is mainly introduces the detailed procedure of encryption and decryption. In Section 4, the experimental results and analysis are given. At the end, we summarize this article.

## 2. Preliminaries

### 2.1. Latin Squares and Transversals

A Latin square of order *n* (defined on an *n*-set *S*) is an n×n array in which each cell contains a single symbol, such that each symbol occurs exactly once in each row and column. For consistency, we set S={0,1,…,n−1}.

Two Latin squares of order *n*$A=(a_{ij})$ and $B=(b_{ij})$ are orthogonal if every ordered pair $\left(a_{ij},b_{ij}\right)$ in S×S occurs exactly once.

**Figure 1:**
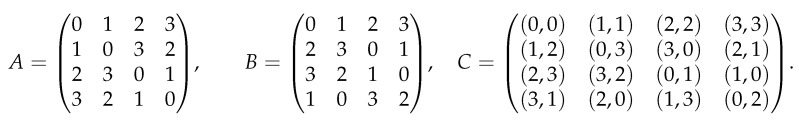
Latin squares *A* and *B* and the juxtaposition array *C*.

Figure 1 lists a pair of orthogonal Latin squares of order 4 $A=(a_{ij})$ and $B=(b_{ij})$. Denote $C=(c_{ij})$ as the juxtaposition array, where $c_{ij}=\left(a_{ij},b_{ij}\right)$. Each ordered pair in S×S occurs exactly once.

Notation: Using a pair of orthogonal Latin squares $A=(a_{ij})$ and $B=(b_{ij})$ can directly generate a two-dimensional map $\phi :\left(i,j\right)\to \left(a_{ij},b_{ij}\right)$,  i,j=0,1,…,n−1.

Suppose *M* is a Latin square defined on *S*. A transversal in *M* is a set of *n* positions, with no two in the same row or column, including each of the *n* symbols exactly once. Two transversals are disjoint if there are no same positions in them. Any *k* disjoint transversal is called a *k*-transversal. If k=n, there exists an *n*-transversal in *M*.

In Figure 1, *C* is the juxtaposition array of *A* and *B*. Treat each column of *C* as a position element set of *A*. There are four positions in the first column; all row numbers and column numbers are different; the four elements at the four positions of *A* are 0,3,1,2 respectively, so the first column of *C* is a transversal of *A*. The other columns of *C* are similar. All the positions of *A* are divided into four pairwise disjoint groups, so there is a four-transversal in *A*.

For an additive group *G*, a bijection θ of *G* is called a complete mapping if the mapping $\sigma :x\overset{\;}{\to }x+\theta \left(x\right)$ is also a bijection of *G* [31].

**Theorem** **1** **([32]).** *The Cayley table M of the additive group $G=\{g_{0},g_{1},\ldots ,g_{n-1}\}$ is a Latin square with the (i,j)th entry $g_{i}+g_{j}$. For a bijection $\theta :G\overset{\;}{\to }G$, $M_{\theta }$ is the Latin square with the (i,j)th entry $g_{i}+\theta \left(g_{j}\right)$, and the cells $\{\left(g_{i},\theta \left(g_{i}\right)\right)\left|i=0,1,\ldots ,n-1\right\}$ form a transversal of M if and only if θ is a complete mapping of G.*

**Theorem** **2.** *Let $F=\{g_{0},g_{1},...,g_{n-1}\}$ be a finite field with character p. M is the Cayley table of F. Let a∈F, a≠0,1, and a≢−1 (mod p). Define a mapping $\gamma_{j}:x\overset{\;}{\to }ax+g_{j}$(j=0,1,…,n−1). Then, the following conclusions hold:*

*(1) These $\gamma_{j}$s (j=0,1,…,n−1) are n different complete mappings over F under addition.*

*(2) Define an n×n array $M_{\gamma }$ with the (i,j)th entry $\gamma_{j}\;\left(g_{i}\right)=ag_{i}+g_{j}$. Then, $M_{\gamma }$ is a Latin square on F.*

*(3) Define $D=(d_{ij})$ with $d_{ij}=\left(g_{i},\gamma_{j}\left(g_{i}\right)\right)$. All columns of D form n disjoint transversals of M (named D as the truncated decomposition array). Define the array $M_{1}$ with the (i,j)th entry $g_{i}+\gamma_{j}\left(g_{i}\right)$. Then, $M,M_{1},M_{\gamma }$ are pairwise orthogonal Latin squares.*

Appendix A shows the proof of Theorem 2. According to this theorem, there are *n* disjoint transversals in *M*, where the *i*th column index in the *j*th transversal is the (i,j)th element of $M_{\gamma }$.

**Example** **1.** *Let F be a finite field of order four. Suppose the primitive polynomial is $\omega^{2}+\omega +1$, where ω is a primitive root of F. Let $F=\{g_{0},g_{1},g_{2},g_{3}\}$ with $g_{0}=0$, $g_{1}=1$, $g_{2}=\omega$, $g_{3}=\omega +1$.*

Firstly, define the Cayley table *M* on the field *F* under addition with the (i,j)th entry $g_{i}+g_{j}$:

$$M=\left(\begin{array}{cccc} 0 & 1 & \omega & \omega +1\\ 1 & 0 & \omega +1 & \omega \\ \omega & \omega +1 & 0 & 1\\ \omega +1 & \omega & 1 & 0 \end{array}\right).$$

Let a=ω. Construct another Latin square $M_{\gamma }$ with the (i,j)th entry $\gamma_{j}\left(g_{i}\right)=ag_{i}+g_{j}$:

$$M_{\gamma }=\left(\begin{array}{cccc} 0 & 1 & \omega & \omega +1\\ \omega & \omega +1 & 0 & 1\\ \omega +1 & \omega & 1 & 0\\ 1 & 0 & \omega +1 & \omega \end{array}\right).$$

Construct the truncated decomposition array *D* with the (i,j)th entry $\left(g_{i},\gamma_{j}\;\left(g_{i}\right)\right)$:

$$D=\left(\begin{array}{cccc} (0,0)\; & (0,1)\; & (0,\omega )\; & \left(0,\omega +1\right)\\ (1,\omega )\; & (1,\omega +1)\; & (1,0)\; & \left(1,1\right)\\ (\omega ,\omega +1)\; & (\omega ,\omega )\; & (\omega ,1)\; & \left(\omega ,0\right)\\ \left(\omega +1,1\right) & \left(\omega +1,0\right) & \left(\omega +1,\omega +1\right) & \left(\omega +1,\omega \right) \end{array}\right).$$

The four positions of each column of *D* form a transversal of *M*, and the set of all columns is a four-transversal of *M*.

Finally, define the array $M_{1}$ with the (i,j)th entry $g_{i}+\gamma_{j}\;\left(g_{i}\right)=\left(1+a\right)g_{i}+g_{j}$:

$$M_{1}=\left(\begin{array}{cccc} 0 & 1 & \omega & \omega +1\\ \omega +1 & \omega & 1 & 0\\ 1 & 0 & \omega +1 & \omega \\ \omega & \omega +1 & 0 & 1 \end{array}\right).$$

According to Theorem 2, $M,M_{1},M_{\gamma }$ are pairwise orthogonal Latin squares.

### 2.2. Logistic Map

In this article, we adopted the classical logistic map to generate two new sequences. One of them was used to generate a finite field, and the other was used to perform diffusion. We describe the logistic map as follows.

(1) $$x_{i+1}=\lambda x_{i}\left(1-x_{i}\right),\;\;\;\;i=0,1,2,\ldots$$

where λ is a system parameter, 0<λ≤4 and $x_{i}\in \left(0,1\right)$. When λ>3.573815, the sequence shows chaos.

## 3. The Proposed Image Encryption Algorithm

**Figure 2:**
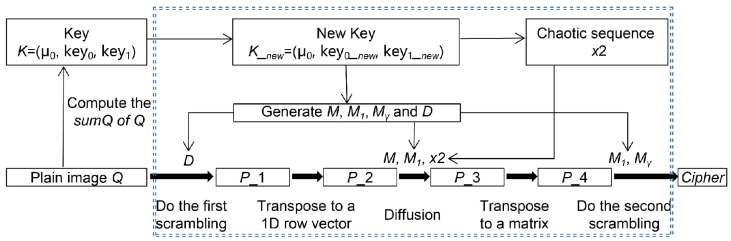
The encryption process.

For simplicity, some of the symbols are described as follows. *n* stands for a prime power. *Q* is used to represent an n×n original plaintext image. *K* is the encryption key. Cipher denotes the corresponding ciphertext. This algorithm is divided into two parts: Algorithm 1 generates three Latin squares and an *n*-transversal by the use of *K* and the features of *Q*; Algorithm 2 is mainly used for encryption, including three layers: scrambling, diffusion, and scrambling, then the encrypted image Cipher is formed. The encryption diagram is listed in Figure 2.

### 3.1. The Generation of Latin Squares $M,M_{1},M_{\gamma }$ and an *n*-Transversal

We used Algorithm 1 to construct three Latin squares and an *n*-transversal, all of which were directly generated on a finite field, using addition and multiplication in the finite field.
**Algorithm 1:** The generation of $M,M_{1},M_{\gamma }$ and an *n*-transversal.Input: An n×n plain image *Q*, encryption key $K=(\mu_{0},key_{0},key_{1})$, public parameter *a*.Output: Latin squares $M,M_{1},M_{\gamma }$ and the truncated decomposition array *D*.Step 1: Compute the sum of all pixels in *Q*, denoted as sumQ. Let
(2)$$s=floor\left(sumQ/255\times 10^{15}\right)/10^{15},$$
where floor is the downward integer function. Compute $key_{0\_new}=\left(key_{0}+s\right)/2$, $key_{1\_new}=\left(key_{1}+s\right)/2$. It is very essential because sumQ reflects the characteristics of the plaintext image. When the plaintext image changes a little, the chaotic sequence will change greatly because of the changed key. In other words, only one round of encryption is needed to achieve a high sensitivity to the plaintext image.Step 2: Generate a logistic sequence of length *n*$x1=\{x_{i}\;\left|\;i=0,1,2,\ldots ,n-1\right\}$ with system parameter $\mu_{0}$ and initial value $x_{0}=key_{0\_new}$. Sort x1 as follows:(3)[fx,lx]=sort(x1),
where sort is the function that sorts a sequence in ascending order. fx is the new sequence reordered by x1, and lx is the index position.Step 3: Redefine the operations of addition and multiplication in lx, then generate a finite field $F_{n}$ with character *p*. Denote $F_{n}=\left\{g_{0},g_{1},\ldots ,g_{n-1}\right\}$. Select $a\in F_{n}$, a≠0,1, and a≢−1(mod *p*), and generate three Latin squares $M,M_{1},M_{\gamma }$ with the (i,j)th entry $g_{i}+g_{j}$, $\left(1+a\right)g_{i}+g_{j}$, and $ag_{i}+g_{j}$, respectively. According to Theorem 2, $M,M_{1},M_{\gamma }$ are pairwise orthogonal.Step 4: Generate the truncated decomposition array *D* with the (i,j)th entry ($g_{i},ag_{i}+g_{j}$). Then, the column set of *D* is an *n*-transversal of *M*.

### 3.2. Image Encryption

**Figure 3:**
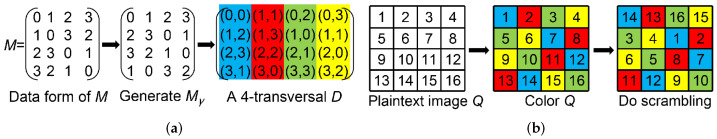
A 4-order example: (**a**) the generation of a 4-transversal *D*; (**b**) the scrambling process according to *D*.

We used Algorithm 2 to complete the rest of the encryption process. First of all, with the help of the truncated decomposition array *D*, we can permutate the image pixels of *Q* group by group. Secondly, we used two Latin squares *M* and $M_{1}$ for auxiliary diffusion based on another chaotic sequence x2. Finally, a pair of orthogonal Latin squares $M_{1}$ and $M_{\gamma }$ was used for the second scrambling. The following is the detailed procedure of Algorithm 2.
**Algorithm 2:** The proposed encryption algorithm.Input: An n×n plain image *Q*, encryption key $K=(\mu_{0},key_{0},key_{1})$, public parameters *a*, $c_{1}$, and $c_{2}$.Output: Ciphertext image Cipher.Step 1: Make use of Algorithm 1, Q,K, and *a* to generate $M,M_{1},M_{\gamma }$, and *D*.Step 2: Scramble *Q* for the first time. At first, convert *D* into a natural column index array $D_{\theta }$ by bijection $\theta :g_{i}\to i$. Starting from the first transversal, the first pixel of *Q* at $D_{\theta }\left(0,0\right)$ is placed at the position $D_{\theta }\left(1,0\right)$, the second pixel at the position $D_{\theta }\left(2,0\right)$ is placed at the position $D_{\theta }\left(3,0\right)$, and so on, until the last pixel at the position $D_{\theta }\left(n-1,0\right)$ is placed at the position $D_{\theta }\left(0,0\right)$. After scrambling *n* times based on *n* transversals, we can obtain a temporary image P_1. The specific process is shown below.
(4)$$\begin{array}{c} \left\{\begin{array}{cc} & P\_1\left(D_{\theta }\left(i+1,j\right)\right)=Q\left(D_{\theta }\left(i,j\right)\right),\\ & P\_1\left(D_{\theta }\left(0,j\right)\right)=Q\left(D_{\theta }\left(n-1,j\right)\right),\\ & 0\leq i\leq n-2,\;\;0\leq j\leq n-1. \end{array}\right. \end{array}$$Figure 3 shows a fourth-order example to illustrate the scrambling process in this step. In Figure 3a, a Latin square *M* (generated on the field of Example 1) is converted into digital form. Select an element a=2, then generate $M_{\gamma }$ with the (i,j)th entry $\left(1+a\right)g_{i}+g_{j}$, resulting in a four-transversal *D*, distinguished by four different colors. All 16 positions of a fourth-order matrix are divided into four pairwise disjoint groups. Because $g_{i}=i$, $D_{\theta }=D$, we can scramble *Q* according to *D*. In Figure 3b, starting from the first transversal, the first pixel ‘1’ at (0,0) is placed at (1,2), the second pixel ‘7’ at (1,2) is placed at (2,3), the third pixel ‘12’ at (2,3) is placed at (3,1), and finally, the fourth pixel ‘14’ at (3,1) is placed at (0,0), as is the scrambling of the other transversals. Because *D* is a four-transversal, the first scrambling can be completed after four times.Step 3: Firstly, convert P_1 into a row vector P_2, then generate another new chaotic sequence of length $n^{2}+100$ with system parameter $\mu_{0}$ and initial value $key_{1\_new}$. To eliminate the effect of the initial value, delete the first 100 digits and the rest form a new chaotic sequence x2. *M* and $M_{1}$ are transposed into row vectors $L_{M}$ and $L_{M1}$, which are used as two pseudo-random sequences for auxiliary diffusion to form a new row vector ${\left\{P\_3\left(i\right)\right\}}_{i=0}^{n^{2}-1}$. The detailed diffusion formula is as follows.
(5)$$\begin{array}{c} \left\{\begin{array}{cc} & b=mod(floor\left(x2\left(i\right)*\left(10^{3}+c_{1}*L_{M}\left(i\right)+c_{2}*L_{M1}\left(i\right)\right)\right),256),\\ & P\_3(i)=P\_2(i)⊕b⊕P\_3(i-1), \end{array}\right. \end{array}$$
where the initial value P_3(−1)=0, *b* is a temporary variable, and mod is the module integer function.Step 4: Transpose P_3 to an array P_4. By using the orthogonality of $M_{1}$ and $M_{\gamma }$, we conducted the second scrambling according to (6), and the final ciphertext image Cipher was obtained.
(6)$$\begin{array}{c} \left\{\begin{array}{cc} & P\_4(M_{1}\left(i,j\right),M_{\gamma }\left(i,j\right))\to Cipher\left(i,j\right),\\ & 0\leq i,j\leq n-1. \end{array}\right. \end{array}$$

### 3.3. Image Decryption

**Figure 4:**
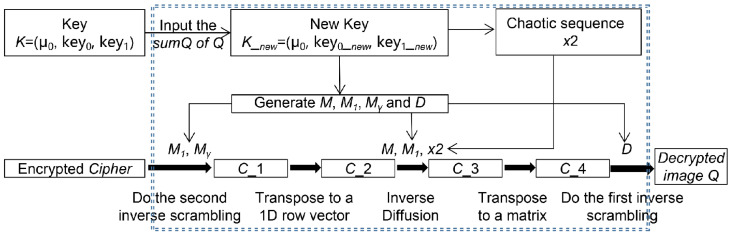
The decryption process.

When we performed image decryption, followed the reverse procedure, and we needed to know the value sumQ in advance. The following Figure 4 is the decryption diagram.

## 4. Simulation Results and Security Analysis

We conducted simulation experiments and list all the results in this section. In order to reflect the superiority of this algorithm, we compared it with some representative algorithms [2,5,24,25,33,34,35,36].

In our experiments, a total of six different 256×256 images were selected for testing, which were chosen from the USC-SIPI2 and CVG-UGR3 image sets. Every experiment required only one round of encryption, and the secret key *K* was: $\mu_{0}$ = 3.99999, $key_{0}$ = 0.123456, $key_{1}$ = 0.234567. There were three public parameters a=ω (ω is a primitive root of $F_{n}$), $c_{1}=1.3,c_{2}=1.5$.

The algorithm was tested from the following aspects: key space and sensitivity analysis, histogram test, correlation test, information entropy test, differential attack resistance test, robustness test, computational complexity, time efficiency analysis, and resistance to classical types of attacks.

### 4.1. Key Space and Sensitivity Analysis

#### 4.1.1. Key Space Analysis

There are three real numbers in $K=(\mu_{0},key_{0},key_{1})$, and the computational accuracy of each value is $10^{-15}$, so this algorithm can achieve a key space of $10^{45}\approx 2^{149}$, greater than $2^{128}$ [37,38]. There are also three public parameters to select, so the algorithm has a large enough key space. In summary, it can resist brute-force attacks.

#### 4.1.2. Key Sensitivity Analysis

An excellent image encryption algorithm desires strong sensitivity to the key, so sensitivity analysis is often considered a crucial indicator of resistance to brute-force attacks. It is usually evaluated from two aspects: sensitivity during encryption and sensitivity during decryption.

**Figure 5:**
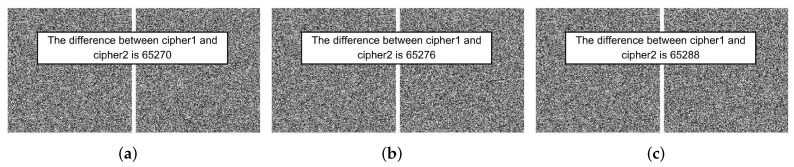
Comparisons of encryption results with key changed. The keys that cipher2 used are: (**a**) $K_{1}=\left(3.99999+10^{-15},0.123456,0.234567\right)$; (**b**) $K_{2}=\left(3.99999,0.123456+10^{-15},0.234567\right)$; (**c**) $K_{3}=\left(3.99999,0.123456,0.234567+10^{-15}\right)$.

(1) Key sensitivity analysis during encryption:

Take the Lena image for example. Firstly, set K=(3.99999, 0.123456, 0.234567), then modify each value slightly by adding $10^{-15}$ after the decimal point. We used two sets of secret keys to encrypt Lena, Cipher1 being the image encrypted with the original key *K* and Cipher2 being the image encrypted with the modified key. Figure 5 shows the comparison of the results of the two ciphertext images. The percentages of different pixels were computed as shown in Table 1, which were all greater than 99.59%, fully indicating that the algorithm is extremely sensitive to the key during encryption.

**Figure 6:**
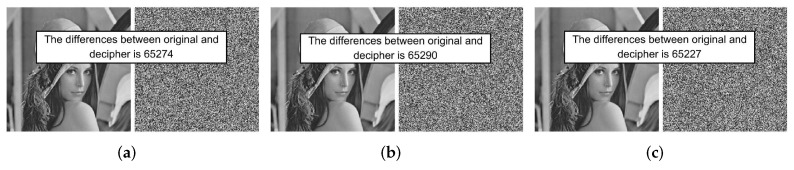
Comparisons of decryption results with the key changed. The keys that decipher uses are: (**a**) $K_{1}=\left(3.99999+10^{-15},0.123456,0.234567\right)$; (**b**) $K_{2}=\left(3.99999,0.123456+10^{-15},0.234567\right)$; (**c**) $K_{3}=\left(3.99999,0.123456,0.234567+10^{-15}\right)$.

(2) Key sensitivity analysis during decryption:

Similarly, Lena was also used to perform key sensitivity analysis during decryption. Given the encrypted image Cipher, make a tiny change to the value $10^{-15}$ in each value of K=(3.99999, 0.123456, 0.234567), then use the two sets of secret keys to decrypt Cipher. From Figure 6, we can find that the original image can only be obtained with the original key, while, when using the modified key, we cannot decrypt correctly. In addition, Table 2 records the percentages of different pixels of two deciphered images, all greater than 99.5%. From these results, we can discover that even though the key changes a little, we will fail to obtain the original image. Therefore this algorithm is key-sensitive during decryption.

### 4.2. Statistical Analysis

A good algorithm for image encryption should be capable of resisting any statistical attacks. The main statistical indicators include histogram analysis, the correlation coefficients of adjacent pixels (usually considering three directions), and information entropy analysis.

#### 4.2.1. Histogram Analysis

In an image, the histogram is a representation of the frequency of each gray-level pixel. A well-encrypted image has as uniform a histogram distribution as possible. In general, it can be measured by variance *S*, and the formula is as follows:

(7) $$S=\frac{1}{256}\sum_{i=0}^{255}{\left(hist_{i}-aver\right)}^{2},$$

where $hist_{i}$ denotes the frequency of the *i*th gray-level pixel, and $aver=\frac{1}{256}\sum_{i=0}^{255}hist_{i}$. *S* represents the variance of the histogram. Set the significance level as α=0.05; if S<293.25, the histogram can be regarded as a uniform distribution [39]. The smaller the value of *S* is, the better.

**Figure 7:**
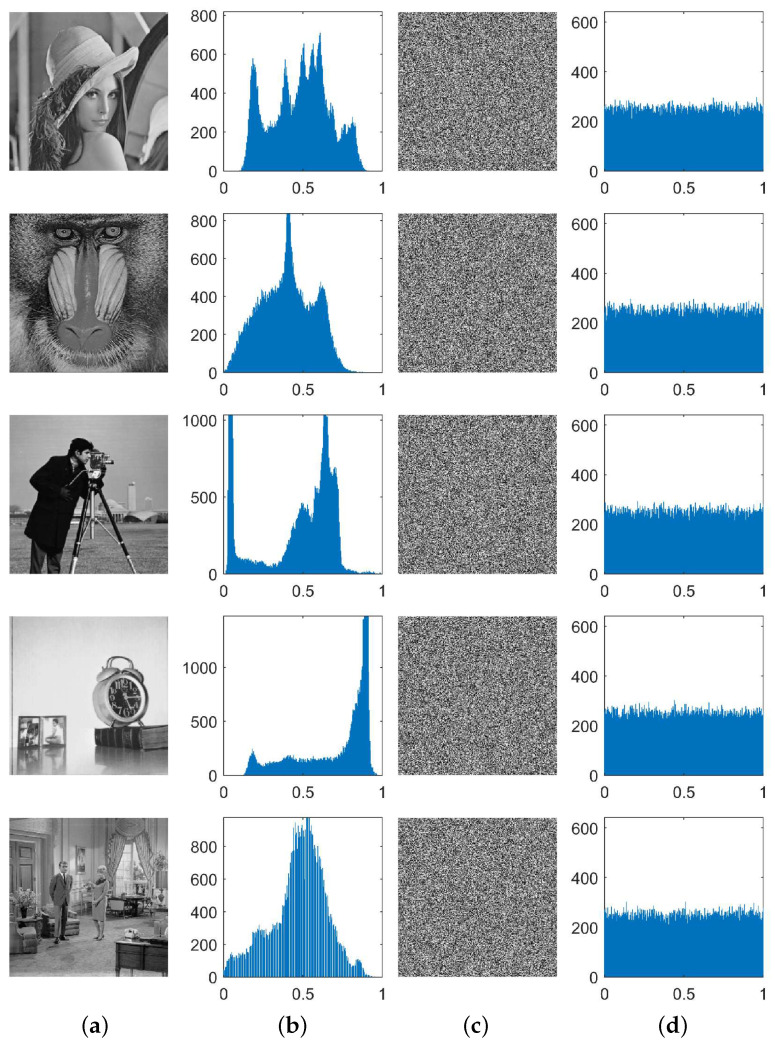
Histograms of “Lena, Baboon, Cameraman, Clock, Couple, Man”: (**a**) plaintext images; (**b**) the corresponding histograms of (**a**); (**c**) ciphertext images; (**d**) the corresponding histograms of (**c**).

Figure 7 shows the histogram distribution of six images before and after encryption. All the histograms of the ciphertext images tend to be evenly distributed. Table 3 shows the histogram values of the six images before and after encryption. All the values of *S* were smaller than 293.25, satisfying the requirements. All encrypted images passed the histogram analysis; especially, the encrypted Lena image’s variance was as low as 195.766. The above results indicate that this algorithm can effectively resist histogram analysis.

#### 4.2.2. Correlation Test

In a plaintext image, there exist strong correlations among adjacent pixels. To resist statistical analysis, the correlation in ciphertext images should be as small as possible [40]. We randomly selected 4000 pairs of neighboring pixels, including three directions (horizontal, vertical, and diagonal) to measure the correlations. The required calculation formula is listed in (8):

(8) $$r_{uv}=\frac{cov(u,v)}{\sqrt{D(u)}\sqrt{D(v)}}.$$

where

(9) $$\begin{array}{c} \left\{\begin{array}{cc} & cov\left(u,v\right)=\frac{1}{N}\sum_{i=1}^{N}\left(u_{i}-E\left(u\right)\right)\left(v_{i}-E\left(v\right)\right)\\ & D\left(u\right)=\frac{1}{N}\sum_{i=1}^{N}{\left(u_{i}-E\left(u\right)\right)}^{2}\\ & E\left(u\right)=\frac{1}{N}\sum_{i=1}^{N}u_{i} \end{array}\right. \end{array}$$

where *u* and *v* represent the grayscale values of two neighboring pixels in the image.

**Figure 8:**
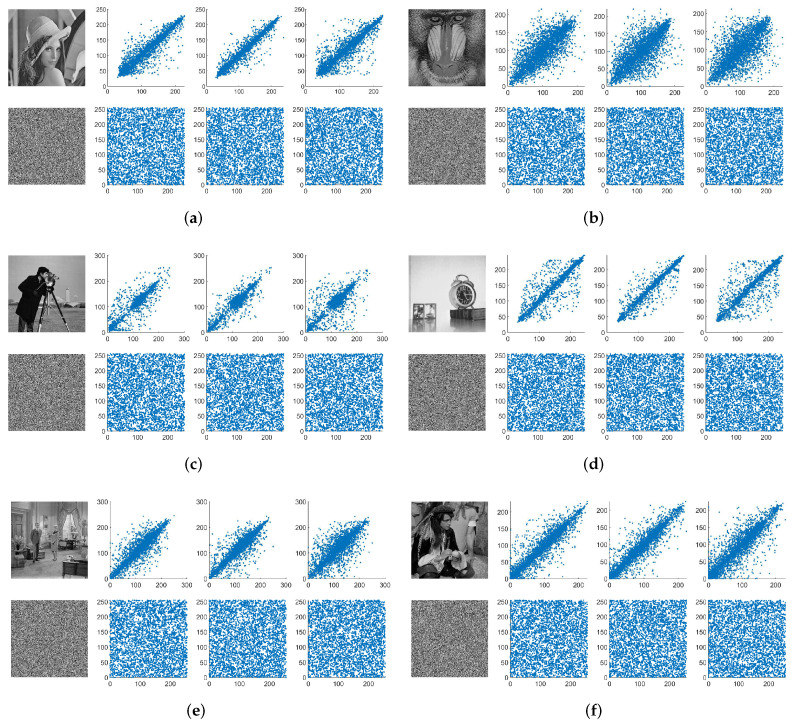
The correlation distribution of plaintext and ciphertext images in the horizontal, vertical, and diagonal directions: (**a**) Lena; (**b**) Baboon; (**c**) Cameraman; (**d**) Clock; (**e**) Couple; (**f**) Man.

To visualize the distribution of the pixels before and after encryption, Figure 8 displays the correlation distributions of six different images in three directions. Observing the original image, we can note that the neighboring dots are mainly distributed around the diagonal, whereas, in an encrypted image, the dots are evenly distributed throughout the whole plane. That is, the plaintext images are highly correlated in any direction, but after encryption, the correlations were very low. Using the calculation formula in [24], we computed the correlation coefficients of six images before and after encryption and present the results in Table 3. We can see that, before encryption, the correlation coefficients were very large, the largest number being approximately 1. However, after encryption, all numerical results were very small, approximately 0. For comparison with other algorithms, Table 4 lists the comparison results in the case of Lena. Although the average correlation coefficient was inferior to [2,33,36], it was better than the other five References. The accuracy of the decimal point was $10^{-3}$, which implies that this algorithm passed the correlation test and achieved a good confusion effect.

#### 4.2.3. Information Entropy Analysis

An important measure of testing randomness is information entropy, usually denoted as *H*, which can be measured according to (10):

(10) $$H\left(m\right)=-\sum_{i=0}^{l-1}p\left(m_{i}\right)log_{2}p\left(m_{i}\right),$$

where $m_{i}$ is the gray value, and there are *l* kinds of gray values in an image. $p(m_{i})$ represents the probability of $m_{i}$, and $\sum_{i=0}^{l-1}p\left(m_{i}\right)=1$. Generally, an image has 256 gray values. Only when the frequency of each gray level is the same, information entropy *H* reaches the theoretical ideal value of 8 [41].

We used (10) to calculate the entropy values of six images before and after encryption, then list the results in Table 3. From the table, we can see that all values were very close to 8, which shows a good encryption effect. Especially, the information entropy of Lena reached 7.99784, and the strong uncertainty of this algorithm was achieved. Table 4 lists Lena’s entropy values in different algorithms. Our result was closest to 8, which was superior to the other contrast algorithms. Therefore, the ciphertext images have strong uncertainty, and our algorithm can resist entropy attacks.

### 4.3. Differential Attack Analysis

A good algorithm can resist differential analysis, requiring different plaintext images (even if with only one different pixel) corresponding to significantly different ciphertext images. In general, there are two commonly used criteria for testing resistance to differential attacks, NPCR and UACI. Let $C_{1}=\left(C_{i,j}^{1}\right)$ and $C_{2}=\left(C_{i,j}^{2}\right)$ denote two ciphertext images of size M×N, where their plaintext image has only one different pixel. Define the binary sequence of the images $C_{1}$ and $C_{2}$:

(11) $$\begin{array}{c} D_{i,j}=\left\{\begin{array}{cc} & 0,\;\;C_{i,j}^{1}=C_{i,j}^{2}\\ & 1,\;\;C_{i,j}^{1}\neq C_{i,j}^{2} \end{array}.\right. \end{array}$$

Then, define NPCR as (12), which means the percentage of different pixels between two ciphertext images.

(12) $$\mathrm{NPCR}=\frac{\sum_{i=0}^{M-1}\sum_{j=0}^{N-1}D\left(i,j\right)}{M\times N}\times 100\%.$$

Furthermore, define UACI as (13), which means the average of the absolute difference between two ciphertext images.

(13) $$\mathrm{UACI}=\frac{\sum_{i=0}^{M-1}\sum_{j=0}^{N-1}\left|C_{i,j}^{1}-C_{i,j}^{2}\right|}{255\times M\times N}\times 100\%.$$

Table 5 shows the encrypted Lena image’s NPCR and UACI at four specific positions, from which we can discover that the values are different at different positions. That is to say, these two indicators have randomness. For unity, let us reduce the first pixel at position (0,0) by 1 and calculate the values of the NPCR and UACI of the six images based on (12) and (13). The numerical results are listed in Table 6.

With a significance coefficient of 0.05, the ideal NPCR is 99.5693%, and the UACI is 33.2824% for images of size 256×256 [42]. Most results in Table 6 were all higher than the expected values, which proved that the algorithm in this article effectively passed the differential attack capability test. For the comparison with other algorithms in the case of Lena, the reader can refer to Table 4. The values of NPCR and UACI were all higher than the contrast algorithms. This means our results were better, indicating our algorithm’s superiority.

### 4.4. Robustness Test

In the process of transmitting ciphertext images over the network, the data may be lost or attacked by noise, which requires the ciphertext image to have good anti-cutting and anti-noise attack performance. In other words, a good algorithm for image encryption should have robustness [43]. In addition, we can use the PSNR to evaluate the quality of the decrypted image and the original image. The higher the value is, the more similar the two images, and the formula (14) is as follows:

(14) $$\mathrm{PSNR}=10\times log_{10}\frac{M\times N\times 255^{2}}{\sum_{i=0}^{M-1}\sum_{j=0}^{N-1}{\left(P\left(i,j\right)-C\left(i,j\right)\right)}^{2}}.$$

**Figure 9:**
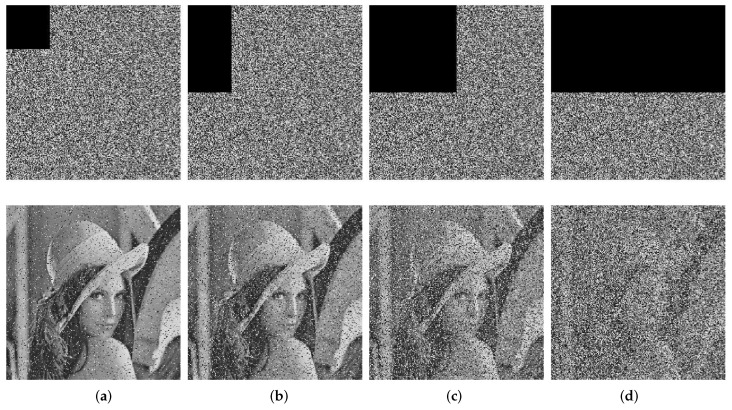
The ciphertext images of cutting off and the corresponding decryptions: (**a**) cut 1/16; (**b**) cut 1/8; (**c**) cut 1/4; (**d**) cut 1/2.

Taking Lena as an example, from the encrypted Lena image, we, respectively, cut off 1/16, 1/8, 1/4, and 1/2 of the data at the top left corner, then decrypted the cut ciphertext images with the correct key. Figure 9 displays the results, which clearly show that, even after its data are cut in half, the body of the image is still visible. The corresponding PSNRs of Figure 9 and other images are shown in Table 7. It is obvious that all PSNR values were larger than 8, which means that this algorithm has a good cutting resistance.

**Figure 10:**
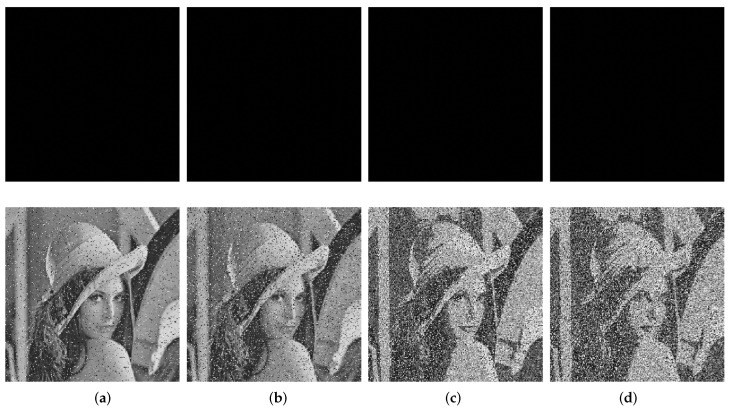
The ciphertext images attacked by different types of noise and the corresponding decryptions: (**a**) salt and pepper noise with density 0.05; (**b**) salt and pepper noise with density 0.1; (**c**) Gaussian noise with variance 0.01; (**d**) Gaussian noise with variance 0.1.

Still taking Lena as an example, we, respectively, used salt and pepper noise with a density of 0.05 and 0.1 and Gaussian noise with a variance 0.01 and 0.1 to attack. As shown in Figure 10, the image is still visible, and the corresponding PSNRs of Figure 10 and other images are listed in Table 7. All PSNR values were larger than 9, indicating a good performance to resist noise attacks.

### 4.5. Computational Complexity and Time Efficiency

Any effective image encryption algorithm requires low computational complexity. In Algorithm 1, one chaotic sequence is generated with computational complexity O(n), and three Latin squares are constructed with computational complexity O$\left(3n^{2}\right)$. In Algorithm 2, there is a three-layer encryption structure, the first scrambling, diffusion, and the second scrambling. The computational complexity is O$\left(n^{3}\right)$, so the computational complexity of this algorithm is O$\left(n^{3}\right)$.

The fast encryption speed of the proposed algorithm can meet the requirements of instant encryption. The experimental environment was MATLAB R2019b, Microsoft Windows 10 with Intel core i5-1135G7, 2.40 GHz processor, and 16 GB RAM. Table 8 shows the encryption and decryption time of the six images, which is the mean value of 20 calculations. We can find that all encryption times were approximately equal to 0.31 s, and the decryption times were approximately equal to 0.27 s. The comparison results of Lena and other algorithms are listed in the Table 4. It can be seen that our algorithm had a relatively faster encryption speed than the other algorithms.

### 4.6. Resistance to Classical Types of Attacks

There are four classical types of attacks: ciphertext only, known plaintext, chosen plaintext, chosen ciphertext. Among them, the chosen plaintext attack is the most powerful attack. If an algorithm can resist this attack, it can resist others [27].

**Figure 11:**
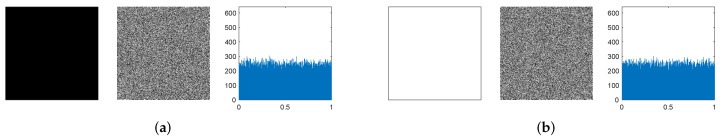
The plain image, encrypted image, and histogram of the encrypted images: (**a**) all-black; (**b**) all-white.

The proposed algorithm only needs one round of encryption to achieve a safe effect. It depends on the plaintext image and is very sensitive to the initial parameters $\mu_{0}$ and initial values $key_{0}$, $key_{1}$. If the plaintext image or one key has changed, $M,M_{1}$, $M_{\gamma }$, and *D* would be totally different. Furthermore, in the diffusion stage, the encrypted value is not only related to the plaintext value and former ciphered value, but also related to the second chaotic sequence. Therefore, the proposed algorithm can resist the chosen plaintext/ciphertext attack, as well as other types of attacks. In addition, hackers often use all-black or all-white images to attack the encryption algorithm. We also performed experiments on these images. The performances are shown in Table 9 and Figure 11.

From Figure 11, the encrypted images became meaningless, and the histograms were uniformly distributed. In addition, from Table 9, we can see that the correlation coefficients were approximately equal to 0.02, the NPCR and UACI were close to the ideal values, and the information entropy was equal to 7.9969. In [15], the information entropy of encrypted all-black and all-white images was 7.9943 and 7.9941, respectively. Therefore, our algorithm performed better. Therefore, the proposed algorithm has the ability to resist the known plaintext attacks and chosen plaintext attacks.

## 5. Conclusions

In this article, a new combinatorial structure was introduced to perform image encryption. An *n*-transversal in a Latin square can not only group all the positions, but also provide a pair of orthogonal Latin squares. The good performance of the *n*-transversal is fully utilized throughout the encryption process. At first, we realized the first substitution group by group according to the *n*-transversal, then we selected two suitable and uniform Latin squares to perform auxiliary diffusion based on a chaotic sequence, achieving good diffusion results. In the end, a pair of orthogonal of Latin squares was made full use of by performing the second scrambling. Three layers of encryption structure were formed. The proposed algorithm successfully encrypted all test images and passed the key sensitivity test, statistical test, plaintext sensitivity test, robustness test, etc. Moreover, the entropy of each encrypted image was very close to 8, and the correlation coefficient was very small, close to 0. From the above analysis, the proposed algorithm had an excellent encryption effect on security and efficiency, outperformed many of the latest papers in terms of some statistical safety indicators, and simultaneously showed robustness and practicability.

This work established the link between the theory of combinatorial designs and image encryption. In the future, we will introduce more combinatorial structures into the image encryption algorithms.

## Figures and Tables

**Table 1:** Key sensitivity test results during encryption.

The Comparison Ciphers	Figure 5a	Figure 5b	Figure 5c
Number of different pixels	65,270	65,276	65,288
Percentage	99.5941%	99.6033%	99.6216%

**Table 2:** Key sensitivity test results during decryption.

Original and Decrypted Image	Figure 6a	Figure 6b	Figure 6c
Number of different pixels	65,274	65,290	65,227
Percentage	99.6002%	99.6246%	99.5285%

**Table 3:** All the results before and after encryption.

Image	Testing Direction			Average Value	Variance	Entropy
	H	V	D			
Lena	0.94034	0.97136	0.92288	0.94486	41,398.1016	7.42489
Ciphertext image of Lena	−0.00064	−0.00356	−0.00157	0.00192	195.7656	7.99784
Baboon	0.78885	0.74049	0.68020	0.73651	46,866.8281	7.37811
Ciphertext image of Baboon	−0.00291	−0.00005	0.00402	0.00233	238.9062	7.99737
Cameraman	0.96099	0.97463	0.92712	0.95425	105,604.8672	7.03056
Ciphertext image of Cameraman	0.00198	0.00045	0.00202	0.00148	227.3594	7.99748
Clock	0.95009	0.97750	0.93230	0.95330	282,061.5625	6.70567
Ciphertext image of Clock	0.00135	0.00365	−0.00194	0.00231	205.8984	7.99775
Couple	0.87446	0.88660	0.80207	0.85438	86,692.7031	7.05625
Ciphertext image of Couple	−0.00067	−0.00159	−0.00023	0.00083	252	7.99723
Man	0.93943	0.95108	0.91287	0.93446	37,058.7812	7.53608
Ciphertext image of Man	0.00347	−0.00098	−0.00128	0.00191	234.7734	7.99741

**Table 4:** Comparison with other algorithms.

Image	Testing Direction			Average Value	Entropy	NPCR (%)	UACI (%)	Encryption Time (s)	Decryption Time (s)
	H	V	D						
Ciphertext image in the proposed algorithm	−0.0006	−0.0036	−0.0016	0.0019	7.9978	99.617	33.5426	0.3077	0.2709
Ciphertext image in [5]	0.0023	0.0158	0.0147	0.0583	–	99.6101	33.4583	0.325	–
Ciphertext image in [24]	0.0179	0.022	7 × 10^−6^	0.0133	7.9970	99.6107	33.4232	0.425	–
Ciphertext image in [25]	0.0018	0.0016	−0.0027	0.002	7.9974	99.6095	33.4649	0.2–0.23	0.13–0.17
Ciphertext image in [33]	0.0009	0.0001	0.0000	0.0003	7.9974	99.6102	33.3915	0.1062	–
Ciphertext image in [34]	−0.0059	−0.0146	0.0211	0.0139	7.9973	99.6100	33.4800	0.3243	–
Ciphertext image in [2]	−0.0003	−0.0007	−0.0001	0.0004	7.9977	99.6000	33.4500	1.3	–
Ciphertext image in [35]	0.0026	0.0051	0.0003	0.0027	7.9973	99.5800	33.5400	–	–
Ciphertext image in [36]	−0.0005	0.0012	0.0008	0.0008	7.9975	99.6037	33.4606	–	–

**Table 5:** Lena’s NPCR and UACI at specific positions (%).

Location	(209,232)	(33,234)	(162,26)	(72,140)
NPCR	99.6353	99.6185	99.6292	99.6246
UACI	33.4139	33.4155	33.416	33.4107

**Table 6:** The NPCR and UACI of the six images.

Image	NPCR (%)	UACI (%)
Lena	99.6170	33.5426
Baboon	99.6307	33.4622
Cameraman	99.6292	33.2594
Clock	99.6124	33.51
Couple	99.6307	33.5925
Man	99.6078	33.3822

**Table 7:** PSNRs with different cutting attacks and noise attacks.

Image	PSNR Values (dB)				PSNR Values (dB)			
	Cut 1/16	Cut 1/8	Cut 1/4	Cut 1/2	Salt and Pepper Noise (0.05)	Salt and Pepper Noise (0.1)	Gaussian Noise (0.01)	Gaussian Noise (0.1)
Lena	18.4952	15.6102	12.7567	10.3489	19.3543	16.5183	13.2037	11.9735
Baboon	18.5505	15.5962	12.7419	10.4122	19.3658	16.2732	13.1195	11.9209
Cameraman	17.6986	14.8126	12.0811	9.6798	18.4388	15.6301	12.2959	11.1939
Clock	16.6901	13.6156	10.7295	8.1282	17.4574	14.4200	11.0870	9.9733
Couple	18.8945	15.8033	12.9758	10.5872	19.4215	16.4731	13.0921	12.0530
Man	17.7603	14.6126	11.7237	9.4005	18.1337	15.3696	11.7741	10.7375

**Table 8:** The encryption and decryption time of the six images.

Image	Encryption Time (s)	Decryption Time (s)
Lena	0.30769	0.27087
Baboon	0.30816	0.27063
Cameraman	0.30946	0.27259
Clock	0.30812	0.27233
Couple	0.30727	0.27219
Man	0.30769	0.27266

**Table 9:** The performances of all-black and all-white images.

Image 256 × 256	Testing Direction			Average Value	Variance	Entropy	NPCR (%)	UACI (%)
	H	V	D					
All-black	−0.00260	0.00045	−0.00286	0.00197	282.6875	7.99688	99.6292	33.2993
All-white	−0.00308	0.00091	−0.00193	0.00197	277.7813	7.99693	99.5728	33.2807

## Data Availability

Not applicable.

## Appendix A. Proof of Theorem 2

(1) Let $F=\{g_{0},g_{1},\ldots ,g_{n-1}\}$ be a finite field with character *p*. Cayley table *M* is a Latin square. The (i,j)th entry is $g_{i}+g_{j}$. By the definition of $\gamma_{j}$, it is easy to see that these $\gamma_{j}$s (j=0,1,…,n−1) are *n* different bijections.

For any x∈F, define the mapping $\sigma_{j}:x\to x+\gamma_{j}\left(x\right)$, j=0,1,…,n−1. Then,

(A1) $$\sigma_{j}\left(x\right)=x+\gamma_{j}\left(x\right)=x+\left(ax+g_{j}\right)=\left(1+a\right)x+g_{j}.$$

Obviously, $\sigma_{j}$ is bijective, then $\gamma_{j}$ is a complete mapping of *F* under addition. By the definition of $\gamma_{j}$, these $\gamma_{j}$s are *n* different complete mappings of *F*.

(2) By the definition of $M_{\gamma }$, it is easy to see that each element of *F* occurs exactly once in each row and column of $M_{\gamma }$. Therefore $M_{\gamma }$ is a Latin square as well.

(3) According to Theorem 1 and the definition of *D*, all columns of *D* form *n* disjoint transversals of *M*.

Firstly, let us prove that $M_{1}$ is orthogonal to *M*. By the definition of $M_{1}$, the (i,j)th entry is $g_{i}+\gamma_{j}\left(g_{i}\right)$. That is,

(A2) $$g_{i}+\gamma_{j}\left(g_{i}\right)=g_{i}+\left(ag_{i}+g_{j}\right)=\left(1+a\right)g_{i}+g_{j}.$$

Because a≠0,1 and a≢−1(mod *p*), $M_{1}$ is still a Latin square different from $M,M_{\gamma }$.

Assuming $M_{1}$ is not orthogonal to *M*, there must exist two positions $\left(i,j\right),\;\left(i^{\prime },j^{\prime }\right)$, where $\left(i,j\right)\neq \left(i^{\prime },j^{\prime }\right)$, such that $\left(g_{i}+g_{j},\left(1+a\right)g_{i}+g_{j}\right)=\left(g_{i^{\prime }}+g_{j^{\prime }},\left(1+a\right)g_{i^{\prime }}+g_{j^{\prime }}\right)$, namely

(A3) $$\begin{array}{c} \left\{\begin{array}{cc} & g_{i}+g_{j}=g_{i^{\prime }}+g_{j^{\prime }}\\ & \left(1+a\right)g_{i}+g_{j}=\left(1+a\right)g_{i^{\prime }}+g_{j^{\prime }} \end{array}\right. \end{array}$$

Then, either $\left(i,j\right)=\left(i^{\prime },j^{\prime }\right)$ or a≡−1(mod *p*). Whichever the case, it is a contradiction with the definition of *a* or the assumption. Therefore, $M_{1}$ is orthogonal to *M*.

Similarly, we can prove $M_{1}$ and $M_{\gamma }$ and *M* and $M_{\gamma }$ are pairwise orthogonal Latin squares. Theorem 2 is proven.
